# Natural language processing of gene descriptions for overrepresentation analysis with GeneTEA

**DOI:** 10.1186/s13059-025-03844-8

**Published:** 2025-10-30

**Authors:** Isabella A. Boyle, Nayeem Akram Aquib, Mustafa Kocak, Randy Creasi, Philip Montgomery, Catarina D. Campbell, Joshua M. Dempster

**Affiliations:** Broad Institute of MIT and Harvard, Cambridge, MA 02142 USA

**Keywords:** Natural language processing, Overrepresentation analysis, Gene sets

## Abstract

Overrepresentation analysis is used to identify biological enrichment in a list of genes. Here, we introduce GeneTEA, a model that ingests free-text gene descriptions and incorporates natural language processing methods to learn a sparse gene-by-term embedding, which can be treated as a de novo gene set database. In benchmarks against existing overrepresentation analysis tools, only GeneTEA properly controls false discovery while consistently surfacing the most relevant biology, doing so with less redundancy. We show that the same approach can be applied to other organisms’ genomes or compounds. Furthermore, we provide an interactive app and API for the trained GeneTEA model.

**Supplementary Information:**

The online version contains supplementary material available at 10.1186/s13059-025-03844-8.

## Background

Technological advances have enabled a shift toward genome-scale, hypothesis-generating experiments. Accordingly, overrepresentation analysis (ORA) was developed to gain biological insights into these high-dimensional data. This method involves testing a query list of genes derived from large-scale experiments for statistical enrichment of gene sets encoding biological processes, molecular functions, phenotypes, or other pre-existing knowledge [1]. There is no universal definition of a gene set, and today they are defined orthogonally across many databases, including the Gene Ontology (GO) [2], Human Phenotype Ontology (HPO) [3], Molecular Signatures Database [4], Kyoto Encyclopedia of Genes and Genomes (KEGG) [5], WikiPathways (WP) [6] and Reactome Database (REAC) [7].

Many tools have been developed to run ORA across these databases simultaneously, with g:Profiler’s g:GOSt [8] and Enrichr [9] among the most popular. However, many issues have been raised with this approach:

- The explosion in gene set databases has resulted in many redundant, conflicting, and poorly defined gene sets [10–12].
- The highly overlapping nature of the gene sets within and between these databases has been shown to reduce the specificity of ORA [13].
- The magnitude of significance values is directly related to the size of the gene set library queried, making it difficult to interpret results from tools that aggregate across many databases [14].
- Many tools suffer from high false discovery rates, often due to improper background definition and underestimation of the number of parallel tests performed [15].

Together, these problems indicate that despite ORA’s widespread adoption, there is still room for improvement.

In recent years, the field of natural language processing (NLP) has experienced many impressive advancements in text representation. Among the simplest NLP models are bag of words (BoW) models, which treat text as a countable collection of unordered tokens (words or phrases); while large language models (LLMs) learn context-dependent embeddings influenced by the position of tokens [16]. Some have proposed LLMs could be leveraged to identify relationships via prompt engineering [17]; for example, TALISMAN [18] and GeneAgent [19] provide tailored prompts for generating names and summaries for lists of genes. However, LLMs are incapable of reporting statistics and are therefore unable to quantify whether the relationships described by their outputs are statistically significant. Furthermore, post hoc steps must be taken to verify that the generative output is not the result of hallucination, a phenomenon where LLMs produce seemingly coherent but factually incorrect outputs. These verification processes measure the semantic similarity of the generated name to the results of ORA on existing gene set databases, effectively restricting these prompting strategies to reporting enrichments that traditional ORA could identify.

A recent comparative study of biomedical reasoning NLP tasks found that LLM performance is highly sensitive to prompt engineering and achieved equivalent results to a simpler BoW model [20]. A potential explanation for this is that LLMs trained on massive corpora scraped from the internet encounter many incomplete, outdated, and conflicting research results [21]. For example, one group performed text-mining on a corpus of more than 200,000 abstracts from dementia publications and found that 91% (325/335) of KEGG pathways were implicated in Alzheimer’s Disease by 5 or more studies [22].

**Fig. 1:**
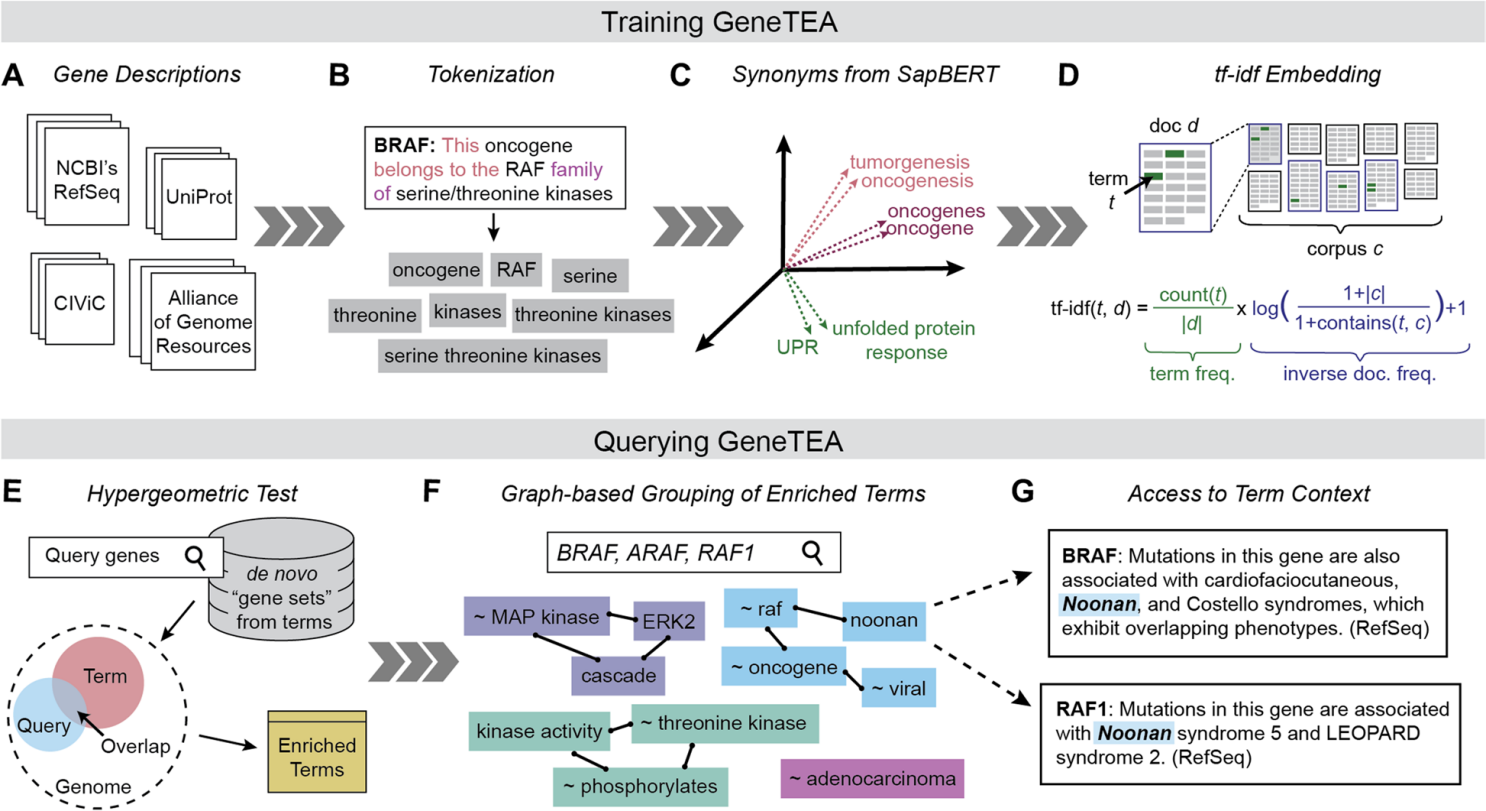
Overview of the GeneTEA model. **A** The training corpus was constructed from free-text gene descriptions. **B** Example of tokenization. **C** Representation of SapBERT embeddings for tokens, colored by the assigned synonym set. **D** Diagram and equation representing term frequency-inverse document frequency (*tf-idf*) embedding. **E** Graphic representing the use of a hypergeometric test to identify enriched terms. **F** Example of the term groups for the query *BRAF*, *ARAF*, *RAF1*. **G** Text excerpts referencing the term “noonan” in *BRAF* and *RAF1*

We hypothesized that NLP could be leveraged to address the shortcomings of ORA by training a BoW model to identify enriched terms in free-text gene descriptions. A similar idea explored by Leong and Kipling [22], which tested whether single-word tokens extracted from PubMed papers could be used for ORA, showed promise. However, this approach preceded many advancements in NLP—leaving room for improvement in surfacing relevant terms, eliminating stopwords (common, uninteresting words), and consolidating synonymous tokens. Furthermore, to ensure the corpus concretely links text to specific genes, we focused on gene descriptions as they distill general knowledge about protein function, localization, and pathway involvement alongside major findings from the literature. Incorporating these concepts, we developed the Gene-Term Enrichment Analysis (GeneTEA) model (Fig. 1). Our novel BoW-based approach learns a gene-by-term embedding from gene descriptions that serves as a compact representation of biological prior knowledge. Critically, GeneTEA does not rely on a priori defined gene sets, avoiding the redundancy and overlap faced by traditional ORA tools that simultaneously query many gene set libraries. Moreover, the unified de novo gene set database underlying our model enables proper background definition and false discovery control. To assess GeneTEA, we analyzed its embedding, benchmarked its performance against competitor ORA tools g:GOSt and Enrichr, and tested the framework’s flexibility in generalizing to other organisms’ genomes and other biological entities such as drugs. Additionally, we provide an interactive app (https://depmap.org/genetea) and API (https://depmap.org/genetea-api/docs) to enable analysis with the pretrained human GeneTEA model.

## Results

### Design of the GeneTEA model

The GeneTEA model was constructed around a corpus of free-text gene descriptions from public resources including NCBI’s RefSeq [23], UniProt [24], Clinical Interpretation of Variants in Cancer (CIViC) [25], and the Alliance of Genome Resource’s natural language summary of the Gene Ontology terms [26] (Fig. 1A). Each text was split into sentences, which were then tokenized into words. Additionally, phrases defined in the UMLS Metathesaurus [27] were extracted to retain only biologically meaningful *n*-grams (Fig. 1B).

We noticed many synonymous terms were present in the vocabulary—such as “oncogene” and “oncogenes” or “unfolded protein response” and “UPR”—and sought to identify them systematically using semantic similarity. Therefore, we clustered token embeddings from SapBERT [28], an LLM fine-tuned on PubMed, using HDBSCAN [29] over pairwise cosine distance (Fig. 1C). We compared this unsupervised synonym set assignment to lists of manually curated concept names specified in UMLS Metathesaurus and found strong agreement between the clusterings (Additional file 1: Figure S1A). These learned synonym sets were labeled by the most frequent term with the prefix “ ~ ” and treated as drop-in replacements for all terms in the cluster.

After texts were preprocessed, a term frequency-inverse document frequency (*tf-idf*) embedding was constructed, such that gene description documents were represented as a gene-by-term sparse matrix (Fig. 1D). The *tf-idf* embedding is a popular BoW representation in NLP, which scales the number of appearances of a term in a document (term frequency, *tf*) by the number of documents containing that term across the corpus (inverse document frequency, *idf*). This normalization up-weights rarity and repetition, attributing higher values to more salient terms. In fact, *tf-idf* values can be interpreted as the mutual information between a term and a document [30]. This gene-by-term matrix could then be binarized to produce a de novo gene set database learned from the corpus of gene descriptions.

When querying GeneTEA, the user provides a list of genes. Per term, the number of genes in the query whose description contains that term is counted and a hypergeometric test is performed to determine overrepresentation (Fig. 1E). This *p*-value is corrected with Benjamini-Hochberg’s method for multi-hypothesis testing to produce a false discovery rate (FDR) value. An FDR threshold of 0.05 is applied to obtain enriched terms and the sum of *tf-idf* values across the query is reported as the effect size, which determines the sort order. Any stopwords or terms matching only a single gene are filtered out, and strict sub-terms are removed if they are less informative than a longer super-phrase.

For queries with strong underlying biological signals, many related—but not strictly synonymous—terms may arise. To address this, manually constructed ontologies such as GO deliberately encode a hierarchical structure to facilitate simplification of enrichment results, for example, via omission of less specific parent nodes. Inspired by this approach, GeneTEA reports term groupings identified via a graph-based strategy that condenses terms with associated meanings in the context of the query (Fig. 1F). Concretely, a graph connecting terms by their similarity in the *tf-idf* embedding is used to identify communities of conceptually related terms. To account for context specificity, the edge weight is the product of the cosine similarity of a pair of terms within the query and across the entire corpus. Low weight edges are pruned, then a greedy-modularity algorithm is used to identify communities. For example, in a query with *BRAF*, *ARAF*, and *RAF1* the terms “ ~ raf”, “ ~ oncogene”, “noonan”, and “ ~ viral” form a group since in this context these describe the RAF families’ initial discovery as a viral oncogene in murine models. These groups are then labeled by the three most informative terms—for example, the term group above would be labeled “ ~ raf |~ oncogene | noonan”.

A critical advantage of GeneTEA’s construction is that the link between the term and source text is retained, allowing a user to refer directly to the context in which that term was used (Fig. 1G). For instance, “noonan” could be a puzzling term to encounter, but by reviewing the text excerpts from the query genes’ descriptions it becomes apparent this term refers to Noonan Syndrome, a disease associated with germline mutations in the RAF and RAS families [31]. In addition to enabling users to assess the reliability of the enrichment, this functionality eases the process of hypothesis building by establishing a direct explanation for how query genes relate to the terms identified.

All versions of the GeneTEA model described in this study were trained locally on a laptop in under an hour with minimal memory usage. However, to facilitate use of the pre-trained human GeneTEA model, we established an interactive app at https://depmap.org/genetea for quick analysis of gene lists. This tool takes in a query of genes, identifies enriched terms and produces simple summary visualizations. The text excerpts for a term in a given gene’s descriptions are easily accessible, with hyperlinks to the source databases. We also provide an API at https://depmap.org/genetea-api/docs such that the model can be programmatically accessed to obtain enriched terms, text excerpts, genes matching a given term, and more.

### Benchmarking approach

We compared GeneTEA head-to-head with g:GOSt and Enrichr to benchmark our ORA model on several tasks. Both of these state-of-the-art models follow the traditional ORA approach of querying many gene set libraries simultaneously. g:GOSt focuses mainly on manually curated ontologies (ie. GO, HPO) and pathway/protein/regulatory motif databases (ie. KEGG, WikiPathways, CORUM [32], TRANSFAC [33]). Enrichr takes an expansive approach, incorporating 130 libraries including ontologies, pathway/protein/regulatory motif databases, as well as gene sets scraped from literature and other bio-knowledgebases. Both competitor models also utilize a hypergeometric test; however, they apply differing multiple hypothesis correction strategies: Enrichr uses Benjamini–Hochberg FDR correction similar to GeneTEA, while g:GOSt employs a simulation-based *p*-value adjustment strategy to limit false discovery to 5%. For a fair comparison, we applied the equivalent FDR threshold of 0.05 to Enrichr and GeneTEA.

### GeneTEA avoids common pitfalls of ORA

**Fig. 2:**
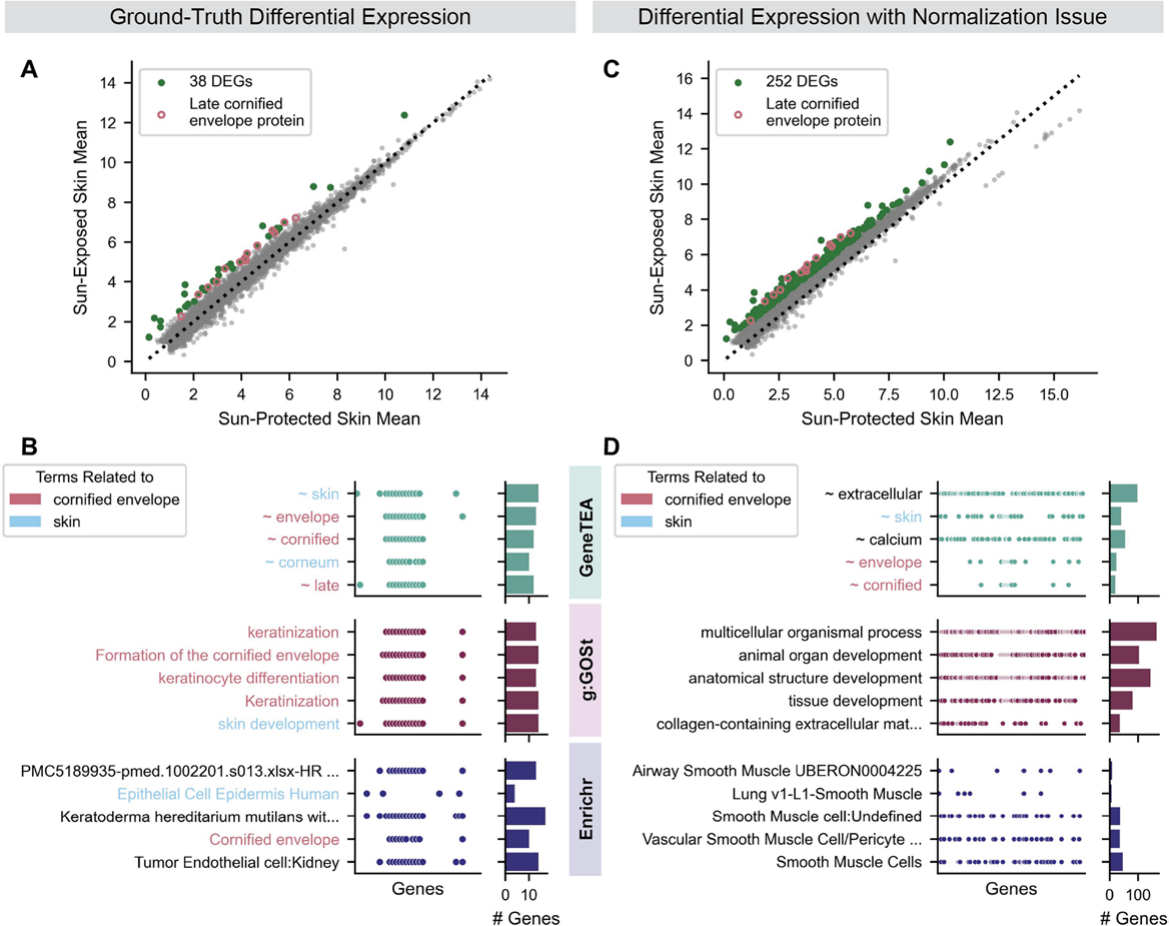
Example of a common ORA use case. **A** Mean expression in log2(RPM + 1) of sun-exposed (*n* = 756) vs sun-protected skin (*n* = 653) samples from GTEx, with differentially expressed genes (DEGs) in green and late cornified envelope proteins from HGNC outlined in pink. **B** Enriched terms recovered by the ORA models when queried with the 38 DEGs highlighted in **A**. Points in the scatter indicate whether a gene on the x-axis belongs to the gene set described by each term on the y-axis, with number of genes a term appears in on the bar plots to the right. **C** Same as **A**, but after introducing a normalization issue by increasing the mitochondrial gene expression in the sun-protected skin samples. **D** Same as **B**, but using 252 DEGs highlighted in **C**

To illustrate the issues with traditional ORA methodology, we conducted an example analysis in which we sought to determine which genes were more highly expressed in sun-exposed lower leg than sun-protected suprapubic skin, leveraging GTEx [34] data from 756 and 653 healthy patients respectively (Fig. 2A). First, we normalized raw counts using reads per million (RPM), and then identified the ground-truth differentially expressed genes (DEGs) with a right-tailed Wilcoxon rank-sums test and mean difference > 1 log2(RPM + 1). We then obtained enriched terms from each of the models (Fig. 2B), all of which surfaced the expected upregulation of the formation of the cornified envelope via keratinization to protect against UV damage [35]. Even in this analysis, Enrichr reported confusing, poorly-defined gene sets scraped from PubMed supplemental tables (“PMC5189935-pmed.1002201.s013.xlsx-HR HER2- Amplification-1Q21 3 1 ERBB2”) and bio-knowledgebases (“Keratoderma hereditarium mutilans with ichthyosis ORPHA:79,395” from Orphanet_Augmented_2021 and “Tumor Endothelial cell:Kidney” from CellMarker_Augmented_2021), confounding the true biological signal.

We then repeated the same analysis, but deliberately introduced a common variety of normalization issue by increasing five-fold the expression of all mitochondrial genes in sun-protected samples prior to the RPM normalization stage, resulting in a noisy set of DEGs (Fig. 2C) [36]. GeneTEA still recovered the “ ~ skin”, “ ~ envelope”, and “ ~ cornified” terms (Fig. 2D). However, the competitor models were unable to identify the interesting underlying biology among the top terms. Instead, they reported enrichment of other non-specific or unrelated terms like “multicellular organismal process” or lung/muscle-related terms that are unlikely to be biologically relevant in a comparison of skin samples.

This analysis exemplifies several common issues encountered with traditional ORA:

- Different libraries often define the same concept, resulting in redundancy.
- Despite manual curation, the hierarchical structure of ontologies often yields overlapping, broad gene sets that mask more specific or informative biology.
- A lack of robustness leading to false positives.

As a consequence, hypothesis generation from methods like g:GOSt and Enrichr can be difficult. In the following analyses, we performed a variety of benchmarking tasks to demonstrate the consistency of these failure modes and show GeneTEA’s systematic improvement over state-of-the-art competitor tools.

### GeneTEA’s embedding efficiently encodes biological prior knowledge

**Fig. 3:**
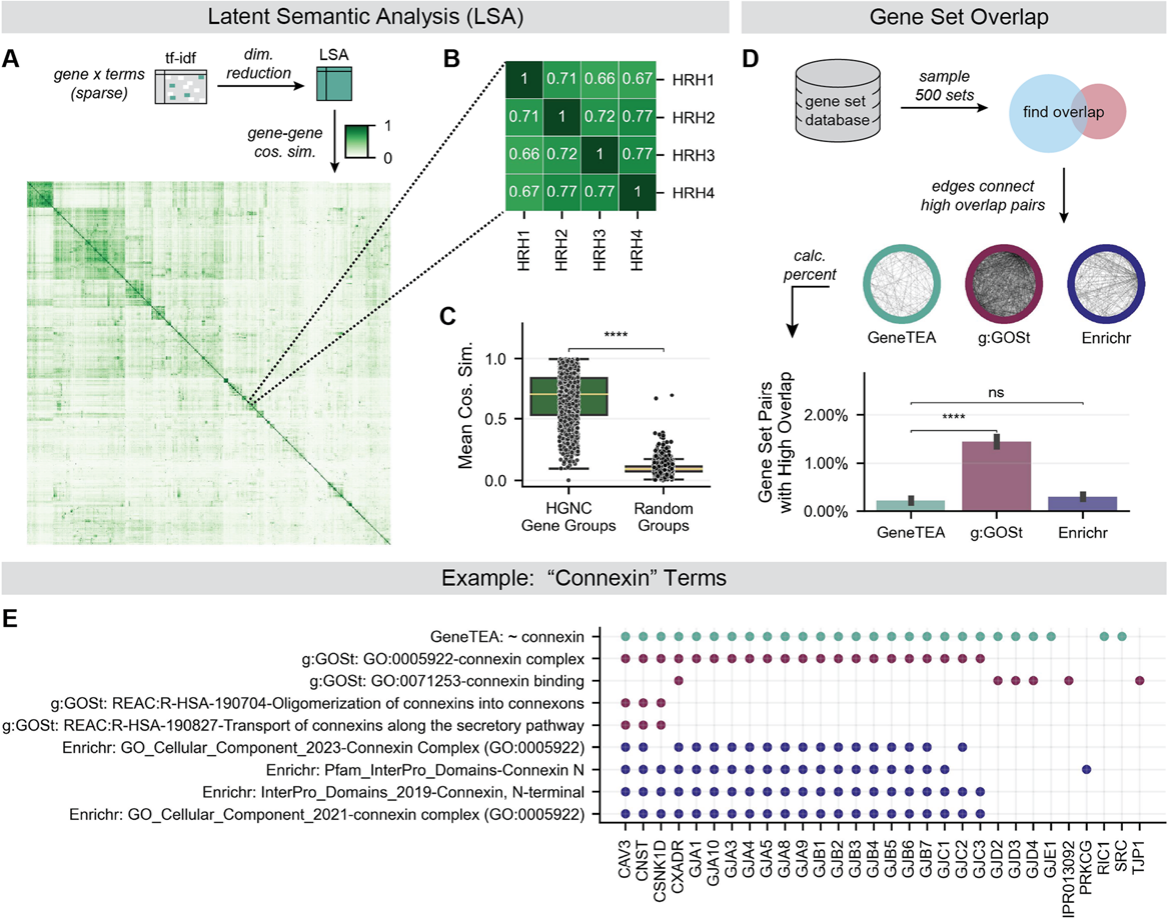
Examination of GeneTEA’s underlying embedding. **A** Upper: Latent semantic analysis (LSA) embedding was produced via a dimensionality reduction GeneTEA’s *tf-idf* matrix. Lower: Clustermap of cosine similarity between protein-coding genes in HGNC groups with < 5 members (*n* = 1,689) in LSA embedding. **B** Cosine similarity of HGNC’s Histamine receptor gene group in LSA embedding. **C** Average cosine similarity for HGNC’s gene groups in LSA embedding, compared to an equal number of random groups of equivalent size (*n* = 1,443). **D** Upper: Hairball plots where nodes are a sample of 500 gene sets from each model’s database and edges represent a high overlap between a pair of gene sets. Lower: Bar plot comparing the mean percent of gene set pairs with high overlap across 10 samples, with error bars representing the standard error of the mean. **E** Example of gene sets from each database related to connexins, where points indicate whether a gene on the x-axis belongs to the gene set on the y-axis. Significance values are from a right-tailed Student’s *t*-test in **C** and a left-tailed Student’s *t*-test in **B**, where **** *p*-value < 0.0001 and ns indicates not significant

To examine the biological signal captured by GeneTEA, we performed latent semantic analysis (LSA) on protein-coding genes to reduce the dimensionality to 500 components. We then evaluated gene–gene similarity by calculating the pairwise cosine similarity over the LSA embedding (Fig. 3A). We observed that gene groups defined by HGNC, for example, the Histamine receptor family of genes, had high average cosine similarity (Fig. 3B-C), which demonstrated that genes defined as related by an orthogonal source were represented by similar term embeddings in GeneTEA.

GeneTEA’s embedding contains ~ 24 k terms (Additional file 1: Figure S1B), which is nearly half the size of g:GOSt’s database (~ 44 k terms) and an order of magnitude smaller than Enrichr’s database (~ 226 k terms). We assessed whether GeneTEA’s smaller embedding contained similar prior knowledge to that which is encoded in manually curated ontologies and pathway databases. To do so, we randomly sampled 250 gene sets from the Gene Ontology, Reactome, and WikiPathways databases and determined if GeneTEA had a semantically similar term with overlapping genes (Additional file 1: Figure S1C). We found that despite its smaller database size, GeneTEA had an equivalent term for 88% of the sets tested (96.4% for GO, 85.6% for REAC, and 82% for WP), indicating it captured similarly precise biology compared to manual annotation. Furthermore, only a small percentage of pairs of gene sets had high overlap in GeneTEA (Fig. 3D). This highlights that conflicting definitions from combining different ontology/pathway resources can lead to many highly overlapping gene sets—a problem GeneTEA avoids as it constructs gene sets from free-text while collapsing synonyms. For example, in GeneTEA’s database, a single term identifies genes related to connexins, while g:GOSt and Enrichr each contain 4 gene sets—various Gene Ontology terms, Reactome pathways, and InterPro domain-containing proteins (Fig. 3E).

### GeneTEA yields reliable and relevant ORA results

**Fig. 4:**
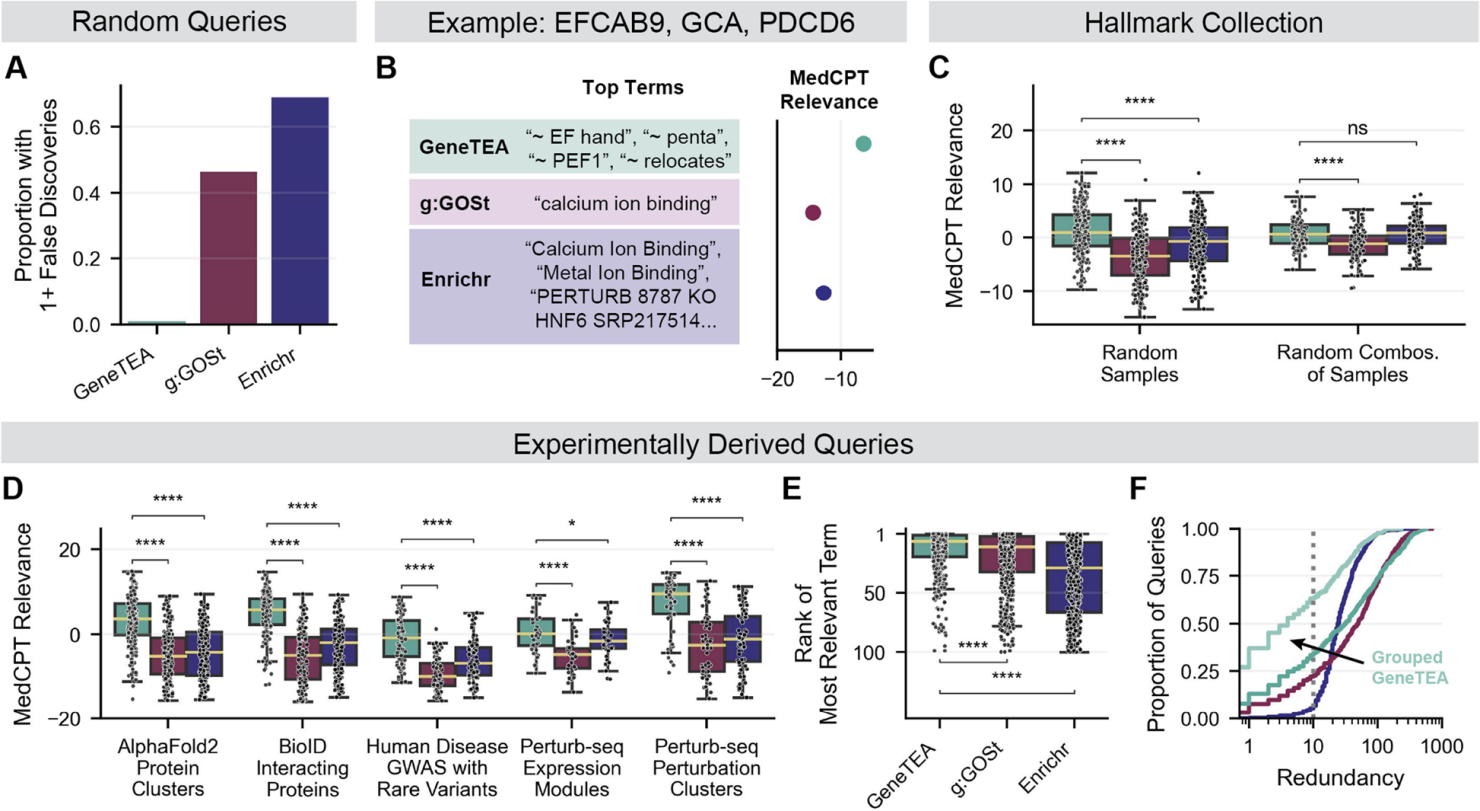
Performance of GeneTEA and competitors on a variety of benchmarking tasks. **A** The proportion of random queries (*n* = 110) that resulted in 1 or more false discoveries. **B** Example of MedCPT Relevance metric. For genes encoding three EF-hand proteins, the top 100 terms from each model are obtained and ranked by MedCPT, resulting in a unitless continuous value where a higher value indicates higher relevance. **C** MedCPT Relevance for random samples of gene sets in the Hallmark Collection (*n* = 200) and random combinations of these samples (*n* = 100). **D** MedCPT Relevance for various experimentally derived queries (*n* = 532). **E** Rank of the most relevant term in a model’s enrichment results for a query, as indicated by highest MedCPT Relevance. **F** Cumulative distribution plot of redundancy (semantically similar term pairs) across experimentally derived queries (*n* = 532). All significance values in **C**-**E** are from a right-tailed Student’s *t*-test, where * *p*-value < 0.05, **** *p*-value < 0.0001, and ns indicates not significant

To test how well GeneTEA and its competitors control false discovery, we queried the models with 110 random gene sets of various lengths between 3–1000 and recorded the number of enriched terms (Fig. 4A). GeneTEA produced false discoveries in only 1 of the random queries tested (< 1%). In contrast, g:GOSt and Enrichr produced false discoveries in 46.4% and 69.1% of random queries, respectively. In total, Enrichr produced 1051 false discoveries while GeneTEA produced only 1, and g:GOSt yielded 116 (Additional file 1: Figure S1D). Enrichr’s false discovery control failed more often in shorter length queries (< 100 genes) while g:GOSt showed the inverse pattern and tended to produce more false discoveries as query length increased (Additional file 1: Figure S1D-E). Furthermore, we found that when tasked with distinguishing gene sets taken from a given model’s underlying database from random sets of genes of equivalent length, GeneTEA outperformed both g:GOSt and Enrichr in terms of F1 Score (Additional file 1: Figure S1F). This was due to GeneTEA’s significantly higher precision, which aligns with its low false discovery rate, as well as perfect recall. Together, these results indicate that GeneTEA is less susceptible to false positives than competitors while maintaining high sensitivity, a crucial characteristic when generating hypotheses from ORA results.

To assess an ORA model’s ability to detect biological signals, we needed a method for measuring the relevance and redundancy of enriched terms. For this purpose, we utilized MedCPT [37], a biomedical information model built to perform lexical matching for semantic retrieval based on 255 million user click logs from PubMed. As MedCPT ranks free-text articles against a user’s search query, it produces a continuous, unitless score for each article where higher values indicate higher relevance for a given search. Therefore, we report this score for the top terms from each model as the MedCPT Relevance (Fig. 4B). For all models, the free-text names of enriched terms were used, rather than solely the semantically meaningless unique identifiers assigned by some ontologies and pathway databases. These term names were concatenated into a period-separated article passed to MedCPT. For example, when GeneTEA was queried with *EFCAB9, GCA, PDCD6*, the enriched terms produced the article “ ~ EF hand. ~ penta. ~ PEF1. ~ relocates.” For g:GOSt, the article was “calcium ion binding” as this was the only enriched term, while Enrichr’s 30 terms produced the article: “Calcium Ion Binding (GO:0005509). PERTURB 8787 KO HNF6 SRP217514 DOWN. Human parvovirus B19 infection… Metal Ion Binding (GO:0046872)…” The MedCPT Relevance was highest for GeneTEA, as the proteins encoded by these query genes have a conserved penta-EF hand domain involved in calcium ion relocation. Critically, this scoring metric quantified that the terms identified by GeneTEA were more specific to the query than the broader categorization of calcium binding captured by the other two models. Additionally, we leveraged the MedCPT article encoder to quantify redundancy in top terms. Specifically, we calculated the number of term pairs with high semantic similarity (cosine similarity of embeddings > 0.95) amongst a given model’s top terms. By this definition, Enrichr’s aforementioned terms contained the redundant pair “Calcium Ion Binding” and “Metal Ion Binding.”

We then tested whether the models recovered relationships in gene sets designed to represent biological signals. For this task, we utilized the Hallmark Collection [38], which contains fifty gene sets representing well-characterized biological states in cancer identified from gene expression patterns across many microarray and RNAseq experiments (Additional file 1: Figure S2A). We randomly sampled 200 queries of sizes 10–100 and found that GeneTEA consistently found terms with higher MedCPT Relevance than the competitors, regardless of query length (Fig. 4C, Additional file 1: Figure S2B-D). We then randomly combined 100 pairs of these sampled queries and found that although the pairs may have contained entirely unrelated sets of genes, GeneTEA still consistently recovered terms with high MedCPT Relevance (Fig. 4C, Additional file 1: Figure S2B-D).

Next, we tested the models on experimentally derived queries where a biological relationship was expected but uncertain (Fig. 4D, Additional file 1: Figure S2E). This included 188 sets of structurally similar proteins identified by clustering AlphaFold2 predictions [39]; 152 sets of interacting proteins identified using proximity-dependent biotinylation (BioID) [40]; 90 sets of genes with rare variants tied to various diseases by genome-wide association studies (GWAS) across the UK BioBank [41]; and finally, 38 expression modules and 64 perturbation clusters where CRISPRi knockdowns produced similar phenotypes in a single-cell Perturb-seq dataset in immortalized cancer cell lines [42]. Again, GeneTEA consistently outperformed other models in identifying terms with higher MedCPT Relevance (Fig. 4D, Additional file 1: Figure S2F-H). When considering the rank order of terms outputted by the models, it was apparent that GeneTEA was better at surfacing the terms with the highest MedCPT Relevance (Fig. 4E). Furthermore, GeneTEA’s top terms had lower redundancy than g:GOSt and Enrichr, especially when utilizing the term grouping strategy (Fig. 4F). While g:GOSt and Enrichr produced more than 10 redundant term pairs in 77.4% and 93.2% of queries respectively, GeneTEA did so in only 65.6% of queries without term grouping and 36.3% with term grouping.

**Fig. 5:**
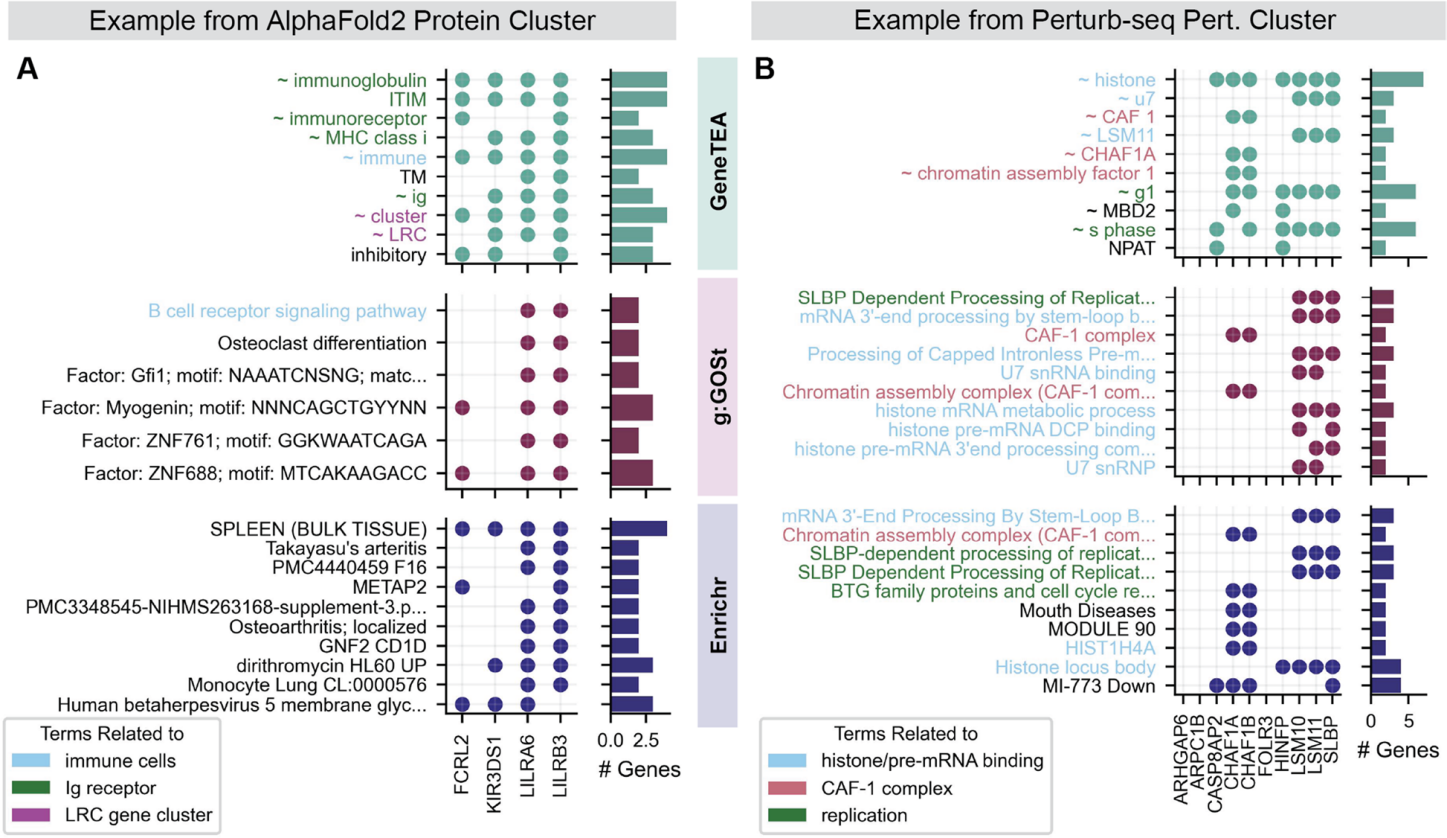
Examples of the top 10 enriched terms from each model. Points in the scatter indicate whether a gene on the *x*-axis belongs to the gene set described by each term on the *y*-axis, with number of genes a term appears in on the bar plots to the right. **A** Top terms for a set of structurally similar proteins identified from AlphaFold2. **B** Top terms for a cluster of Perturb-seq perturbations inducing similar transcriptional phenotypes

One example of a structurally similar protein cluster identified with AlphaFold2—*FCRL2*, *KIR3DS1*, *LILRA6*, and *LILRB3*—highlighted GeneTEA’s ability to identify the biological signal underlying a query (Fig. 5A). The top terms identified by GeneTEA indicated these genes encode immunoglobulin-like receptors that contain immunoreceptor tyrosine-based inhibitory motifs (ITIM), which are critical for binding MHC class I molecules [43]. As this query was constructed by assessing protein structural similarity, it was not surprising that the GeneTEA enrichment emphasized this shared domain. Additionally, the “ ~ LRC” and “ ~ cluster” terms captured that 3 of the 4 genes encode members of the leukocyte receptor complex (LRC) cluster present on the 19q13 cytoband. The competitor models failed to detect the conserved motif and LRC cluster membership, only finding that *LILRA6* and *LILRB3* are involved in B cell receptor signaling.

Another example that emphasized the utility of GeneTEA as a hypothesis-building tool was a cluster of perturbations identified in the Perturb-seq dataset (Fig. 5B). All three models identify that Chromatin Assembly Factors I (CAF-1) genes *CHAF1A*/*CHAF1B* and *SLBP*/*LSM10*/*LSM11* interact with histones during the cell cycle—the former are histone chaperones that enable nucleosome assembly for DNA replication [44] and the latter are involved in processing replication-dependent histone mRNA [45]. However, only GeneTEA identified that *CASP8AP2* and *HINFP* also have replication-related histone function—*CASP8AP2* was shown to regulate the expression of replication-dependent histone mRNA during S phase in colon cancer [46], while *HINFP* is a transcriptional activator that promotes the expression of histone H4 genes at the G1/S phase transition [47]. This bolsters a “guilt by association” hypothesis that *CASP8AP2* and *HINFP* are functionally related to CAF-1 and *SLBP* as they produce a similar transcriptional phenotype following knockdown in this Perturb-seq dataset.

### Underlying GeneTEA is a flexible and extensible framework

**Fig. 6:**
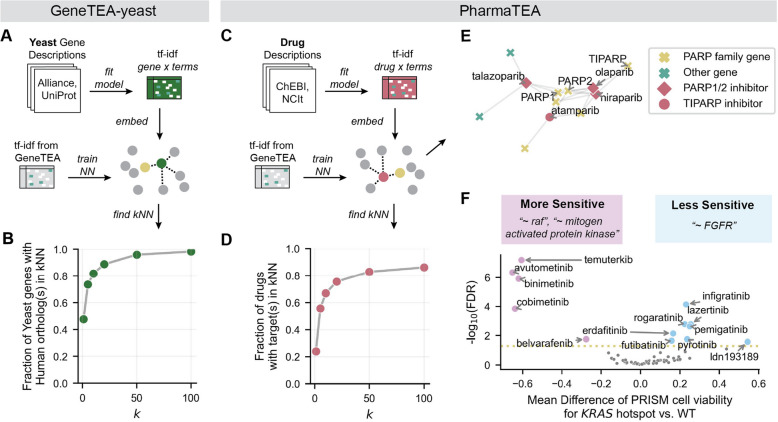
GeneTEA’s flexible framework generalizes to other domains. **A** Schema for training GeneTEA-yeast from a corpus of yeast gene descriptions, then finding its *k* nearest neighbor (*k*NN) GeneTEA (human) genes. **B** Fraction of human orthologs recovered in *k*NN for yeast genes (*n* = 212) at various *k* values. **C** Schema for training PharmaTEA from a corpus of drug descriptions, then finding its *k*NN GeneTEA genes. **D** Fraction of target genes recovered in *k* nearest neighbors for PRISM drugs (*n* = 151) at various *k* values. **E** Spring layout of a graph with cosine distance weighted edges connecting 5 nearest neighbor genes to PARP inhibitors. **F** Top terms for drugs (*n* = 64) that *KRAS* mutant cell lines (*n* = 159) are more or less sensitive to than *KRAS* WT cell lines (*n* = 749), based on two-tailed Student’s *t*-test of the difference in cell viability in the PRISM repurposing dataset

While GeneTEA was initially established to perform ORA on human genes, its framework can be repurposed to identify enrichment across any entity for which a reasonably large corpus of descriptions can be obtained. As a proof-of-concept, we trained GeneTEA for the budding yeast (*Saccharomyces cerevisiae*) genome utilizing text from the Alliance of Genome Resources and UniProt (Fig. 6A). We compared the GeneTEA-human and GeneTEA-yeast *tf-idf* matrices under the assumption that orthologous genes should have similar embeddings. To do so, we trained a *k* Nearest Neighbors (*k*NN) model on the GeneTEA-human *tf-idf* matrix and then queried it with yeast ortholog’s *tf-idf* embeddings (Fig. 6B). This *k*NN model had access to all overlapping terms, except for any term representing a protein-coding gene symbol, as this could achieve high performance trivially. For 212 high-confidence human-yeast orthologs identified by the Alliance of Genome Resources, 47.6% of yeast genes had a human ortholog as their nearest neighbor (*k* = 1), and 95.8% had a human ortholog within their fifty nearest neighbors (*k* = 50). These results demonstrated that GeneTEA could recover shared signals between evolutionarily conserved genes despite being trained on species-specific corpora.

We further hypothesized that the GeneTEA framework could extend to biological entities other than genes, for instance, small molecules or therapeutic compounds; therefore we obtained drug descriptions from Chemical Entities of Biological Interest (ChEBI) [48] and the National Cancer Institute Thesaurus (NCIt) [49] and trained a new model, PharmaTEA (Fig. 6C). For 151 drugs annotated with PubChemCIDs and genetic targets in the Cancer Dependency Map (DepMap) portal, we repeated the *k*NN analysis comparing GeneTEA to PharmaTEA under the assumption that a drug’s nearest neighbors should contain its annotated genetic target(s). While 23.8% had an annotated target as the nearest neighbor (*k* = 1), this improved to 55.6% for the 5 nearest neighbors (*k* = 5) (Fig. 6D). For example, poly-ADP ribose polymerase (PARP) inhibitors are connected to genes in the PARP family in the 5-NN graph (Fig. 6E).

One use case for PharmaTEA is interpreting sensitivity profiles generated in the PRISM repurposing project, for which a variety of FDA-approved compounds were screened across pooled cancer cell lines in an attempt to identify anti-cancer activity [50]. When comparing the viability of 159 cell lines with *KRAS* hotspot mutations to 749 wild-type cell lines across 64 drugs in the PRISM repurposing project, PharmaTEA revealed that those harboring mutations were more sensitive to drugs targeting the RAF family and mitogen-activated protein kinases (MAPKs) than the fibroblast growth factor receptor (FGFR) family (Fig. 6F). Oncogenic *KRAS* is upstream of the RAF family and MAPKs, which explains why inhibition of these targets lead to a viability effect. In contrast, *KRAS* and growth factor receptor mutations like *FGFR* and *EGFR* are generally mutually exclusive in cancer, resulting in a lack of sensitivity to the latter in the *KRAS* hotspot population [51].

## Discussion

Identifying the biological signal underlying a list of genes is a common task; however, the success of ORA hinges upon the queried gene set database. With GeneTEA, we demonstrated that a de novo gene set database could be learned from free-text gene descriptions using NLP to reduce redundancy while retaining specificity and interpretability. While traditional gene set databases require extensive manual curation, GeneTEA was designed to be updated and expanded automatically. This methodology enabled the integration of multiple bio-knowledgebases, producing harmonized results with reliable statistics. Moreover, we showed that GeneTEA’s framework is extensible to other organisms' genomes or other biological entities such as drugs, so long as a corpus of reliable descriptions can be obtained.

GeneTEA produced fewer false positives while consistently surfacing more relevant enriched terms versus competitor ORA tools. This is likely due to the compact, less overlapping, and more homogeneous nature of the gene sets database in GeneTEA. Methodological improvements including proper false discovery control, ranking terms based on information content, and filtering redundant terms offered additional benefits over competitors. These results demonstrated that GeneTEA yields reliable and specific enrichment results that enable rapid hypothesis generation.

Furthermore, GeneTEA’s *tf-idf* matrix encoded known relationships between genes. Future work could explore using this embedding in semi-supervised computational biology analyses where prior knowledge of gene function would be beneficial—for example when defining gene programs from expression data or for feature selection in predictive models. Additionally, it may be interesting to examine the utility of GeneTEA’s *tf-idf* matrix for predicting new gene function annotations from other data modalities such as gene expression, sequence similarity, or protein structure.

## Conclusions

In this paper, we introduced GeneTEA, which leverages NLP of gene descriptions to identify interpretable, reliable, and relevant biological signal overrepresented in lists of genes. We demonstrated that the gene-by-term embedding efficiently encodes prior knowledge of gene function. Finally, we provide an API (https://depmap.org/genetea-api) and interactive analysis app (https://depmap.org/genetea) to facilitate use of the pre-trained human GeneTEA model; as well as an open-source code base that enables this framework to be applied to other genomes and biological entities (https://github.com/broadinstitute/GeneTEA) [52].

## Methods

### Human gene description corpus

To assemble our corpus, we obtained free-text Human gene descriptions from the following sources:

- Alliance: Downloaded *Homo sapiens* gene descriptions from version 8.0.0 (Accessed 2025–02–21) [26]
- CIViC: Downloaded Features TSV from 01-Feb-2025 Release (Accessed 2025–02–21) [25]
- NCBI: Obtained RefSeq summaries from https://ftp.ncbi.nlm.nih.gov/gene/DATA/gene_summary.gz, as well as names/aliases and gene locations in https://ftp.ncbi.nlm.nih.gov/gene/DATA/GENE_INFO/Mammalia/Homo_sapiens.gene_info.gz (Accessed 2025–02–21) [23]
- UniProt: Downloaded Function, Domain, Subunit structure, etc. sections for all Reviewed (Swiss-prot) Human proteins (Accessed 2025–02–21) [24]

### Tokenization and phrase extraction

All gene descriptions in the corpus were mapped to a consistent set of gene symbols using the HGNC symbol mapping [53]. Texts were tokenized into sentences using nltk’s sent_tokenize (v3.7) and into words by splitting on spaces. Tokens were converted to lowercase if only one letter was capitalized, otherwise their original case was retained. Candidate phrases were identified using the UMLS2024AB MRCONSO file (accessed 2025–02–24) [27], where English phrases for each concept defined by at least 2 sources were extracted from sentences.

### Synonym set definition

Each token is embedded with SapBERT via the huggingface API (transformers v4.46.3) based on code adapted from Hu et al. 2023 [54]. First, the AutoTokenizer encoded the input into tokens which were then passed to the AutoModel, followed by mean pooling using the attention mask. These embeddings were aggregated into a matrix, and then sklearn’s HDBSCAN (v1.4.0) was run with min_sample_size = 2 and min_cluster_size = 2 on the embeddings. All clusters extracted were treated as synonym sets named by the most frequent member of the set with the prefix “ ~ ”.

We compared the learned synonym sets to lists of concept names identified in the English portion of the UMLS2024AB MRCONSO file (accessed 2025–02–24) [27]. Concretely, we applied the same tokenization used in GeneTEA to the concept names in the STR column of the file and retained only those overlapping with the GeneTEA vocabulary. We then grouped these concept names by the concept unique identifier (CUI) and retained the 18,780 CUIs containing at least 2 concept names. Finally, we found 12,318 GeneTEA synonyms with at least 2 tokens overlapping the UMLS concept names and computed the homogeneity, completeness, and V-measure clustering scores for these 89,830 tokens with sklearn’s homogeneity_completeness_v_measure function (v1.4.0).

### Embedding with *tf-idf*

We fit an sklearn Tfidfvectorizer (v1.4.0) on the tokenized documents, with the following hyperparameters: max_df = 0.6, min_df = 3, binary = False.

### Statistical tests and effect sizes for overrepresentation

Overrepresentation in GeneTEA is measured using a hypergeometric test. The scipy.stats [55] implementation hypergeom.sf with loc = 1 was used to obtain *p*-values, which were then converted to FDR values using Benjamini-Hochberg’s false discovery control (scipy.stats.false_discovery_control). Version 1.11.1 of scipy.stats was used for this computation. The reported “Effect Size” is the sum of *tf-idf* values per term across the query genes. Additionally, the sum of *tf-idf* values across all genes in the matrix is reported as the “Total Info.”

### Custom stopwords

We found that uninteresting, non-biological terms occasionally appeared enriched—such as “exhibits” or “several”—and used the following strategies to exclude them. First, we took the average SapBERT embedding of the top 500 most frequent words in the 2024 MEDLINE 1-g set [56] and added any terms in the vocabulary with a high Pearson correlation (> 0.65). Then, we added phrases made up entirely of stopwords. From this list of stopwords, we identified synonym sets made up of > 50% stopwords. Finally, we added terms that appeared in > 15% of genes. Any fully uppercase terms or those matching the chromosomal location patterns were not included in this list of custom stopwords.

### Graph-based filtering and grouping strategy

The relationship between query genes and enriched terms can be represented as a bipartite graph, where top nodes are genes and bottom nodes are terms with un-weighted edges representing the presence of a term in a gene’s description. Therefore, we leveraged existing implementations of several graph algorithms from the Python package networkx (v3.2.1) [57] to filter out strictly redundant terms and group conceptually similar terms.

First, a bottom node projection of terms is created where edges are present if a pair of terms is present in a fully overlapping set of genes. Then, edges are pruned such that only sub-terms (i.e. “olfactory” and “olfactory receptor”) remain connected. All terms are then ranked by "Effect Size", "FDR", "Total Info", and finally lexicographically. Finally, term nodes are pruned if the term is not the highest ranked amongst its connected nodes, resulting in a filtered set of terms.

Next, terms are grouped based on the intuition that terms with similar meanings will have similar *tf-idf* embeddings within a query and across all genes. Therefore, a new graph is created where nodes are filtered terms and edges have weight

$$w(t1,t2)=cossim(v(q,t1),v(q,t2))\ast cossim(v(g,t1),v(g,t2))$$

where *v* is the *tf-idf* vector over query genes *q* or all genes *g* for terms *t1, t2* and *cossim* refers to cosine similarity*.* If *w* < 0.15, the edge is removed to prevent collapsing largely unrelated terms. Then, groups of terms are identified using the networkx greedy_modular_communities function and are labeled by the three highest-ranked terms.

### Querying competitor ORA models

For g:GOSt, we obtained human gene set enrichment results from all available libraries via the gprofiler python package’s profile function (v1.0.0). To filter to the top terms, we retained only entries matching at least 2 query genes annotated as significant as determined by the provided adjusted *p*-value threshold. Significance defined the sort order. In cases where the same term was reported in the output multiple times, we retained only the row corresponding to the first appearance.

For Enrichr, we queried the “enrich” API endpoint (on 2025–03–27) for each tested library and concatenated the results. We used the 130 libraries presented on the Enrichr website’s “Transcription”, “Pathways”, “Ontologies”, “Diseases/Drugs”, and “Cell Types” tabs as of 2025–03-03. We retained only entries matching at least 2 query genes where the “Adjusted *p*-value” was below 0.05 and sorted by “Combined score.” In cases where the same term was reported in the output multiple times, we retained only the row corresponding to the first appearance.

### Differential expression analysis of GTEx skin samples

For the example analysis, we obtained GTEx expression data for all protein-coding genes for sun-exposed skin (gene_reads_v10_skin_sun_exposed_lower_leg.gct.gz) and sun-protected skin (gene_reads_v10_skin_not_sun_exposed_suprapubic.gct.gz) [34]. In the version of the analysis with a normalization issue, we multiplied reads of the mitochondrial genes by 5. Then, we applied the reads per million normalization, with a log2-transformation and pseudo-count of 1: log2(reads/sum(reads) * 1e6 + 1). Next, we ran a right-tailed Wilcoxon ranked-sum test (scipy.stats.ranksums) to obtain *p*-values, followed by FDR correction (scipy.stats.false_discovery_control). Finally, we used a significance threshold of 0.01 and a mean difference threshold of 1 log2(RPM + 1) to identify differentially upregulated genes.

### Latent semantic analysis

To better understand the underlying biological signal in GeneTEA’s embedding, we performed latent semantic analysis (LSA) using sklearn (v1.4.0) [58]. First, the *tf-idf* matrix was reduced to 500 dimensions using TruncatedSVD decomposition with ARPACK solver. Then, the matrix was normalized using Normalizer. Finally, pairwise cosine similarities were computed along the gene axis.

### Comparison to manually curated databases

To assess whether GeneTEA contained similar terms to those appearing in manually curated databases, we sampled 250 random gene sets from the GO, Reactome, and WikiPathways libraries present in the g:GOSt database. For each gene set, we computed the semantic similarity between its name and GeneTEA terms with at least one gene in common. Here, semantic similarity was defined as the cosine similarity between MedCPT [37] QEnc ("ncbi/MedCPT-Query-Encoder") embeddings. Only the 250 GeneTEA terms with the highest *tf-idf* sum for the gene set were considered to limit computational complexity. The maximum semantic similarity was reported between a given gene set’s name and the matching GeneTEA terms, as well as the maximum similarity when compared to an equivalent number of randomly sampled GeneTEA terms. A manually curated gene set was considered to have an equivalent in the GeneTEA database if it had a maximum semantic similarity greater than 75% of maximally similar randomly sampled GeneTEA terms.

### Gene set overlaps

To assess the overlap between gene sets within a database, we computed the probability of two randomly selected gene sets having high overlap. Specifically, if the smaller gene set in the pair had at least half its members in common with the larger gene set it was considered highly overlapping. We estimated the probability of high overlap by randomly sampling 500 gene sets from each of the gene set databases underlying GeneTEA, g:GOSt, and Enrichr and found the percent of pairs with high overlap. This process is repeated 10 times to assess stability.

### Assessment of false discovery control

We created a benchmarking set of random queries to assess false discovery control. We randomly sampled lists of 3, 5, 10, 15, 20, 25, 50, 100, 250, 500, and 1000 genes 10 times each, resulting in 110 queries. We then counted the number of enriched terms identified by each model for each query, representing false discoveries.

### Assessment of sensitivity

We designed a simple classification task to assess the ORA models’ sensitivity. First, we queried each model with a sample of 25 real gene sets (with length < 100 genes) from their underlying databases, as well as random sets of genes of equivalent size. Each set was classified as ‘real’ if enriched terms were found and ‘random’ if no terms were significantly enriched. The classifications were then used to compute the precision, recall, and F1 score with sklearn’s precision_recall_fscore_support (v1.4.0) for the ‘real’ class. We repeated this process 5 times to assess stability.

### MedCPT relevance and redundancy metrics

To quantify the relevance and redundancy in a model’s enriched terms, we turned to MedCPT [37]. We utilized the huggingface API (transformers v4.46.3) to access NCBI’s MedCPT CrossEnc ("ncbi/MedCPT-Cross-Encoder") and AEnc ("ncbi/MedCPT-Article-Encoder"). For each tested gene set, we treated the comma-separated list of genes as the query and either a single term or the period-separated top 100 enriched terms from each model as the set of documents. To measure MedCPT Relevance, we embedded the pairs of query and document with the CrossEnc’s tokenizer, then fed this to the model to obtain the output logits. We took the top 100 enriched terms from each model, embedded them with the AEnc, and counted the number of pairs with cosine similarity > 0.95 to quantify redundancy.

### Query gene sets used for benchmarking

To create random samples of the Hallmark Collection [38], we sampled lists of genes of length 10, 25, 50, and 100 from each of the 50 gene sets to create 200 queries. We then randomly combined pairs of these sampled sets to create an additional 100 queries.

To create a set of experimentally derived queries, we obtained lists of genes with length > 2 from the following sources:

- “AlphaFold2 Protein Clusters”: Data file “3-sapId_sapGO_repId_cluFlag_LCAtaxId.tsv.gz” from Barrio-Hernandez et al. 2023 [39] downloaded from https://afdb-cluster.steineggerlab.workers.dev/. UniProt IDs were mapped to gene symbols using the HUGO Gene Nomenclature Committee (HGNC) mapping [53], and only clusters representing > 2 HGNC gene groups were retained.
- “BioID Interacting Proteins”: Supplementary Table 4 of Go et al. 2021 [40]
- “Human Disease GWAS with Rare Variants”: Supplementary Table 8 of Wang et al. 2021 [41]
- “Perturb-seq Expression Modules” and “Perturb-seq Perturbation Clusters”: Supplementary Table 3 of Replogle el al. 2022 [42]

### Training GeneTEA-yeast and PharmaTEA

Free-text yeast gene descriptions for GeneTEA-yeast were obtained from the following sources:

- Alliance: Downloaded *Saccharomyces cerevisiae* gene descriptions from version 8.0.0 (Accessed 2025–02–24) [26]
- UniProt: Downloaded Function, Domain, Subunit structure, etc. sections for all Reviewed (Swiss-prot) *S. cerevisiae* proteins (Accessed 2025–02–24) [24]

Free-text compound descriptions for PharmaTEA were obtained from the following sources:

- ChEBI: Downloaded Record Descriptions (Compound) from PubChem PUG View API (Accessed 2025–02–25) [59]
- NCIt: Downloaded Record Descriptions (Compound) from PubChem PUG View API (Accessed 2025–02–25) [59]

Both models were trained identically to GeneTEA, except that GeneTEA’s synonym sets were used to ensure alignment of term definitions.

### *k *Nearest Neighbors analysis

To compare GeneTEA-yeast and PharmaTEA embeddings to GeneTEA, we ran a *k* Nearest Neighbor (*k*NN) analysis. First, overlapping terms were identified; though terms that matched the protein-coding gene symbols were excluded as they could trivially connect neighbors. The *tf-idf* embedding from GeneTEA was then used to train an sklearn NearestNeighbors model (v1.4.0) with metric = “cosine”. The *k*NN of each yeast ortholog or drug were then obtained for *k* = 1, 5, 10, 20, 50, and 100, and the number of human orthologs/genetic targets at each *k* value was counted. Yeast-human orthologs were obtained from Alliance of Genome Resources (version 8.0.0, accessed 2025–02–21) “Alliance combined orthology data” TSV [26], filtered to only ortholog pairs identified by all 9 algorithms. Drug-target annotations were obtained from the PortalCompounds.csv in the DepMap 24Q4 Public Figshare [60].

### PRISM repurposing sensitivity for KRAS mutant vs. wild-type cell lines

We obtained the PRISM repurposing log2-fold change data, where cell viability was measured as the difference in PRISM barcode abundance between treatment and DMSO conditions, from the file Repurposing_Public_24Q2_Extended_Primary_Data_Matrix.csv in the Repurposing Public 24Q2 Figshare [61]. Using hotspot mutation calls from the OmicsSomaticMutationsMatrixHotspot.csv file on the DepMap 24Q4 Public Figshare [60], we performed a two-tailed Student’s *t*-test (scipy.stats.ttest_ind) between the populations with and without a *KRAS* mutation for the 137 drugs where PubMed CIDs and targets had been annotated. We applied Benjimini-Hochberg false discovery control (scipy.stats.false_discovery_control), then called significant compounds based on an FDR threshold of 0.05, with directionality assigned based on the mean difference between the *KRAS* hotspot and WT cell lines. Lists of significantly more sensitive and less sensitive drugs were used to query PharmaTEA, where the background was specified as the 137 drugs on which the *t*-test was performed. Version 1.11.1 of scipy.stats was used throughout this analysis.

## Supplementary Information

Additional file 1: Supplementary Figures S1-S2.

## Data Availability

The datasets supporting the conclusions of this article are available on Figshare [62]. GeneTEA’s open-source Python codebase (under 2-Clause BSD license) can be found on GitHub at https://github.com/broadinstitute/GeneTEA [52], with the version used in this article archived on Figshare [63].
