# Supplementary material for: Natural language processing of gene descriptions for overrepresentation analysis with GeneTEA
Source: Genome Biol. 2025 Oct 30;26:376. doi: 10.1186/s13059-025-03844-8 (PMC12577433; doi:10.1186/s13059-025-03844-8)
Supplement: Supplementary file 1 — Additional file 1: Supplementary Figures S1-S2. [file 13059_2025_3844_MOESM1_ESM.pdf]

**Supplementary Figures for "Natural language processing of gene descriptions for overrepresentation analysis with GeneTEA"**

Isabella A Boyle[1*]

Nayeem Akram Aquib[1]

Mustafa Kocak[1]

Randy Creasi[1]

Philip Montgomery[1]

Catarina D Campbell[1]

Joshua M Dempster[1]

[1] Broad Institute of MIT and Harvard, Cambridge, MA, 02142, USA

* To whom correspondence should be addressed. Email: iboyle@broadinstitute.org

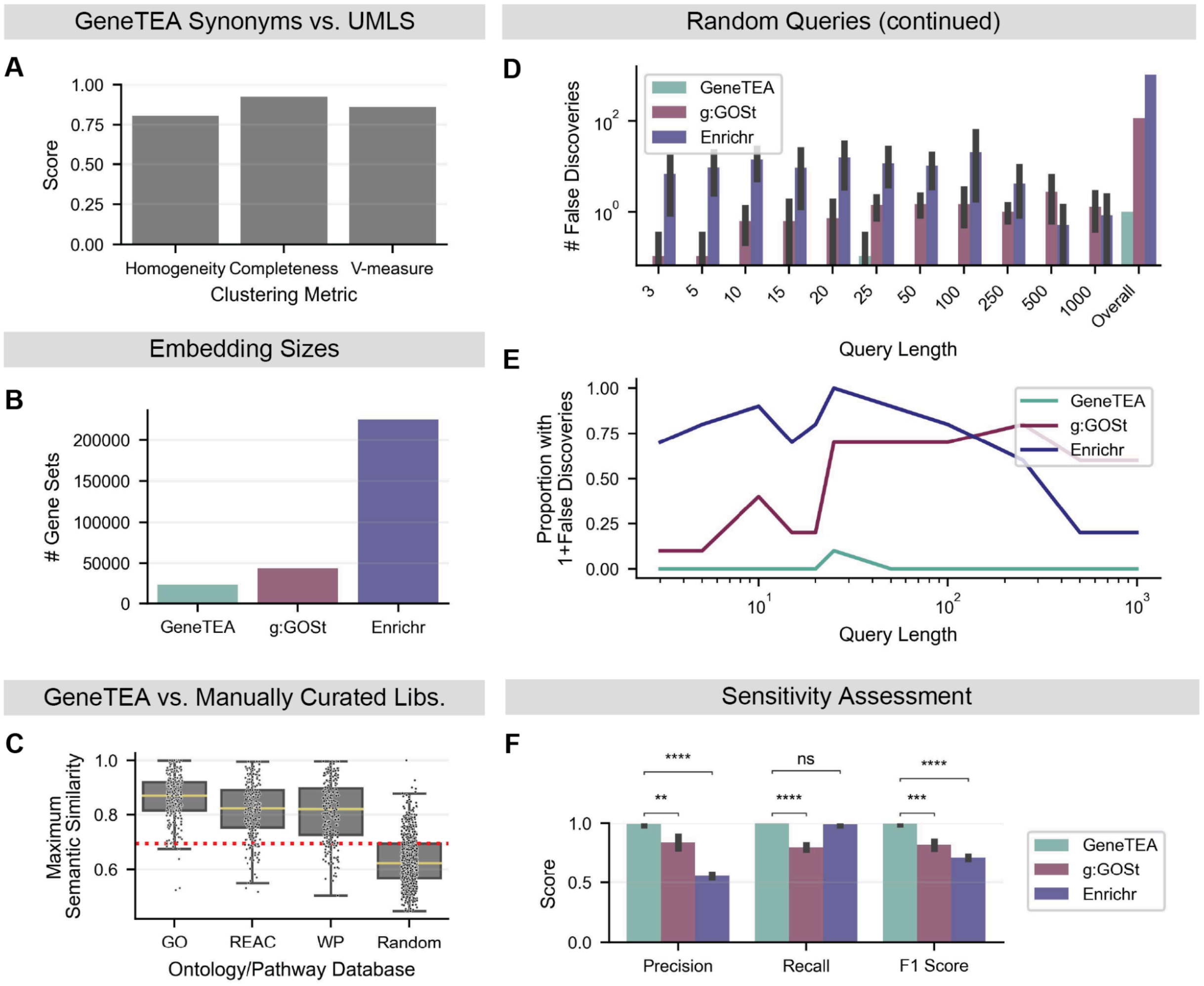


**Figure S1.** Additional information on embedding construction and false discovery control. (**A**) Scores from various clustering metrics when comparing GeneTEA's synonym sets to UMLS Metathesaurus concepts (*n*=89,830 tokens). (**B**) Number of gene sets in each model's embedding. (**C**) Maximum semantic similarity score between random gene set names (n=250) sampled from Gene Ontology (GO), Reactome (REAC), and WikiPathways (WP) databases, and GeneTEA terms with at least 1 gene overlapping. "Random" represents the same comparison, but for a random set of GeneTEA terms. (**D**) Bar plot showing the number of false discoveries for all lengths of queries and overall, with error bars representing the standard error of the mean over 10 samples. (**E**) Proportion of random queries with false discoveries versus length of query. (**F**) Precision, recall, and F1 scores when distinguishing real gene sets from a given model's database (*n*=25) and random gene sets of equivalent size (*n*=25), with error bars representing the standard error of the mean over 5 repetitions. All significance values in **F** are from a right-tailed Student's *t*-test, where ** *p*-value < 0.01, *** *p*-value < 0.001, **** *p*-value < 0.0001.

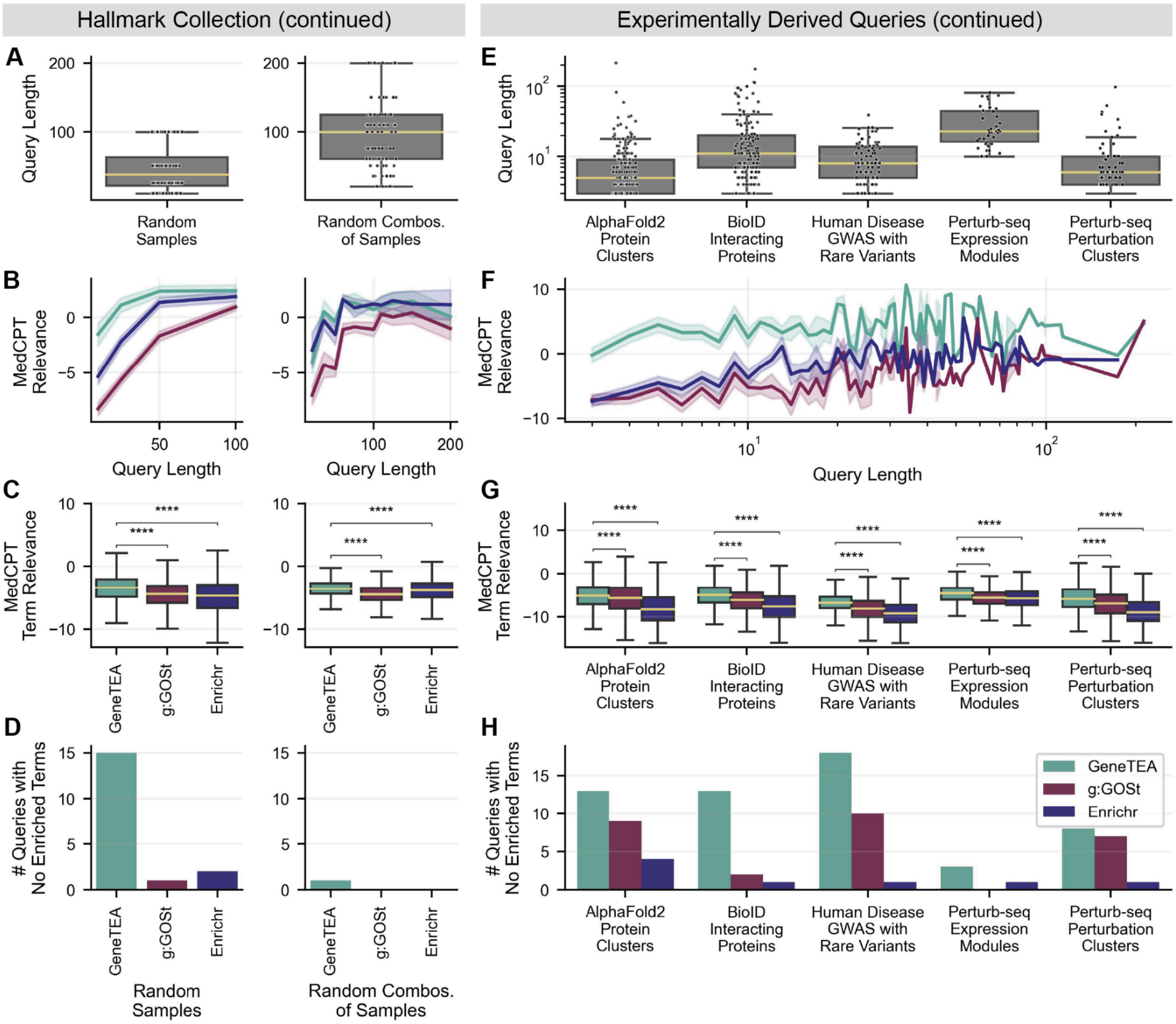


**Figure S2.** Additional information on benchmarking tests. (**A**) Lengths of queries in Hallmark Collection (*n*=200 in left, *n*=100 in right). (**B**) MedCPT Relevance versus length of query in the Hallmark Collection, with error bars representing the standard error of the mean. (**C**) MedCPT Relevance for each Hallmark Collection query's top 100 enriched terms. (**D**) Number of queries where no enriched terms were detected in the Hallmark Collection. (**E**) Lengths of queries in experimentally derived queries (*n*=532). (**F**) MedCPT Relevance versus length of query in experimentally derived queries, with error bars representing the standard error of the mean. (**G**) MedCPT Relevance for each experimentally derived query's top 100 enriched terms. (**H**) Number of experimentally derived queries where no enriched terms were detected in experimentally derived queries. All significance values in **C** and **G** are from a right-tailed Student's *t*-test, where **** *p*-value < 0.0001.
